# Natural Frequencies of Diatom Shells: Alteration of Eigenfrequencies Using Structural Patterns Inspired by Diatoms

**DOI:** 10.3390/biomimetics9020085

**Published:** 2024-01-31

**Authors:** Simone Andresen, Selina K. Linnemann, Ahmad Burhani Ahmad Basri, Oleksandr Savysko, Christian Hamm

**Affiliations:** Alfred Wegener Institute Helmholtz Centre for Polar and Marine Research, Am Handelshafen 12, 27570 Bremerhaven, Germany

**Keywords:** biomimetics, bulging, combs, eigenfrequency maximization, lightweight design, ribs, voronoi, thickness optimization

## Abstract

Diatoms have delicate and complex shells showing different lightweight design principles that have already been applied to technical products improving the mechanical properties. In addition, diatom inspired structures are expected to significantly affect the vibration characteristics, i.e., the eigenfrequencies. Directed eigenfrequency shifts are of great interest for many technical applications to prevent undesired high vibration amplitudes. Therefore, numerous complex diatom inspired dome structures primarily based on combs, ribs, and bulging patterns were constructed and their eigenfrequencies were numerically studied. Different structural patterns were identified to significantly affect eigenfrequencies. The results were compared to dome structures equipped with rib patterns in combination with a common structural optimization tool. The study indicates that a combination of (1) selecting diatom inspired structural patterns that strongly affect eigenfrequencies, and (2) adapting them to the boundary conditions of the technical problem is an efficient method to design diatom inspired lightweight solutions with high eigenfrequencies.

## 1. Introduction

In the large field of biomimetics and bio-inspiration in connection with innovative structural design and structural optimization, biomimetic and bio-inspired structures have already been a very effective source of inspiration for innovative lightweight structures, e.g., [1,2,3,4]. Structural patterns in nature are often complex and irregular, as material is only placed in area where it is needed. Natural structures are the result of different load cases affecting the system. Thus, structural adaptation has been necessary to ensure the survival of the organism.

In regard to the numerous published studies that dealt with optimized lightweight structures inspired by nature, static load cases are very common, e.g., [1,5]. However, a very delicate and important field in structural engineering is how to deal with vibration problems. Structures get excited by external vibrations and start to vibrate in such a way that the functionality is impaired and it can even result in structural damage. Especially lightweight structures are often susceptible towards vibrations due to the reduced weight and the delicateness of the structures. Typically, constructions are employed with dampers or structures are designed in a more massive way to avoid unwanted vibration amplitudes. However, these traditional methods are not suitable for the lightweight technology as they increase the mass and thus contradict the overall goal of using lightweight structures.

**Figure 1:**
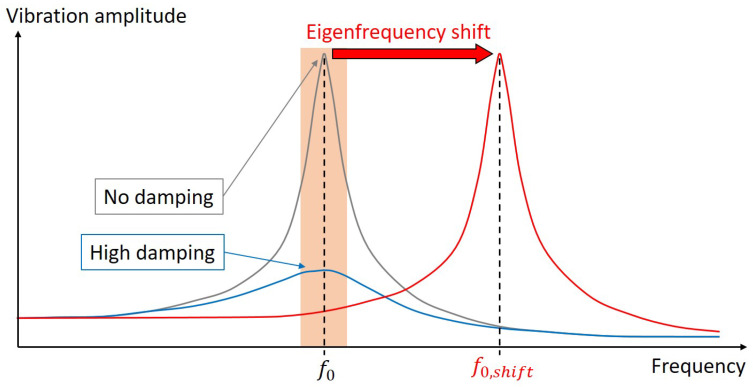
Schematic view of the vibration amplitude depending on the frequency. If the eigenfrequency of the system $f_{0}$ lies within the frequency range of excitation (orange), large vibration amplitudes occur that can be decreasing with damping devices. Shifting $f_{0}$ to the value $f_{0,shift}$ detunes the system in a way that the system’s eigenfrequency does no longer match with the excitation frequencies and high vibration amplitudes are prevented.

Generally, each structure capable of vibrating can be described by its eigenfrequencies (natural frequencies) and its eigenmodes. If a structure gets excited, e.g., due to external vibrations, it vibrates in the shape of one of its characteristic eigenmodes at a characteristic frequency, i.e., the eigenfrequency connected to that specific eigenmode. A coincidence of the frequency of the external vibration with one of the structure’s eigenfrequencies is called ‘resonance’, It can lead to very high vibration amplitudes and therefore to structural damage. Thus, it is very crucial to avoid these resonance phenomena. A promising method to reduce vibration amplitudes in lightweight structures is to adapt the structural design. Changes in geometry ‘detune’ the system and thus alter the eigenfrequencies as illustrated in Figure 1. As a consequence, the structural eigenfrequencies do not match anymore the frequencies of external vibrations and resonance can be avoided.

**Figure 2:**
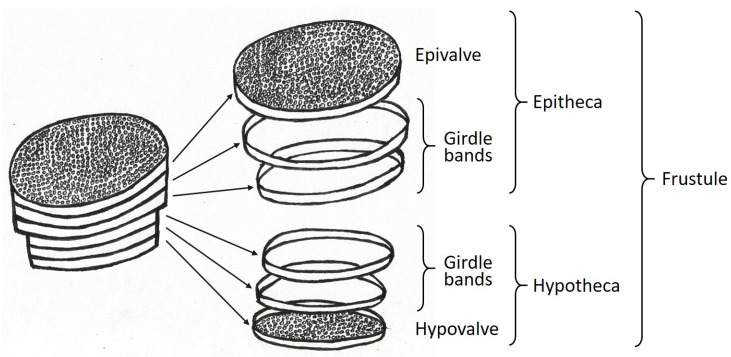
General structure of a diatom shell composed of an epivalve, girdle bands, and a hypovalve.

Lightweight structures inspired by planktonic organisms (diatoms, radiolarians) seem to strongly increase structural eigenfrequencies. Studies showed that especially irregular combs and lattice structures present in diatoms can lead to high eigenfrequencies [6,7,8]. Diatoms are unicellular aquatic algae that possess delicate and complex shells primarily made of silicate. Figure 2 illustrates the general composition of a diatom characterized by an epivale and an hypovalve both connected via girdle bands. The about 20,000 [9] to 200,000 [10] estimated recent diatom species show very diverse shell structures. Diatoms need to float in the sunlight flooded (euphotic) upper water column since sinking into the deep water would lead to death. Therefore, their silicate shells are very light, although silica is far heavier than water. At the same time, the shells have to resist attacks by predators like copepods which take the algae into their feeding tools and try to crack them. Thus, the combination of low weight and high mechanical resilience results in lightweight design principles visible in diatom shells, which are a valuable source of inspiration for designing optimized and innovative lightweight structures across different engineering sectors.

Aside from the lightweight design principles, diatom shells seem also to be a very interesting inspiration for vibration optimized structures. Copepods feeding on diatoms do not only try to crack their prey, but also handle them like a jackhammer [11]. Thus, there are vibrational load cases affecting the algae that have to be survived. While there have been several experimental studies on diatom shells focusing on static load cases (e.g., [12,13,14]) or crushing tests [15], it is very difficult to experimentally investigate the vibrational characteristics of diatoms. Hence, construction and simulation tools common in engineering have been utilized to further study diatoms. Numerous numerical studies focused on the mechanical properties of diatom frustules, e.g., [14,15,16,17]. However, the vibrational characteristics of diatoms have been less often investigated. First experimental studies on the vibrational eigenmodes of diatoms using atomic force microscopy have been carried out [18]. The 1st eigenfrequencies of the diatom shells were between 1–8 MHz. In addition, the effect of diatom frustule density, stiffness, dimensions, pore size, and wall thickness on the eigenfrequencies and mode shapes was analyzed [19]. They simulated very simplified centric and pennate diatoms and obtained 1st eigenfrequencies of about several MHz to tens of MHz. Another study simulated simple diatom shell models stating that diatom shells are already pre-deformed according to eigenmodes indicating that there seem to be vibrational load cases affecting the development of the complex structural patterns present in diatom shells [20]. Further studies showed that pre-deforming structures according to eigenmodes strongly increases the corresponding eigenfrequencies [21]. Thus, this diatom-inspired method to adapt the geometry without increasing the mass appears already to be an efficient method to shift eigenfrequency and to consequently prevent resonance phenomena.

However, as already mentioned, there are numerous different diatom shells varying in their complex structural patterns. Little is known about how these different structural patterns influence the eigenfrequencies. Though, discovering innovative bio-inspired structures that have a strong impact on eigenfrequencies is of great interest for many technical applications, especially in the lightweight design sector.

Therefore, different diatom-inspired dome structures were studied with respect to their eigenfrequencies and eigenmodes. While published studies dealt only with very simplified diatom shells [19,20], more complex structures were designed here. It was focused on diatoms showing different comb structures, rib patterns, and bulging (embossing) structures, as published studies indicated those structural elements to strongly alter eigenfrequencies, e.g., [8,22,23,24]. The resulting complex structures were then compared to optimized structures equipped with diatom-inspired combs. The conducted thickness optimizations aimed at finding the optimal thickness distribution of the dome structure [25].

In total, the presented work is divided into two parts. In the first part, different dome structures inspired by diatoms were designed and numerically studied regarding their eigenfrequencies and eigenmodes. The second part contained the optimization of dome structures equipped with different rib structures inspired by diatom combs. The dome structures resulting from both study parts were then compared among each other. The overall objective was to find structural patterns or design principles that strongly affect eigenfrequencies.

## 2. Materials and Methods

In the following, the design and simulation processes as well as the subsequent optimizations are described. All studies were conducted using the software Rhinoceros (version 6 SR9, Robert McNeel & Associates, Seattle, WA 98103, USA) in combination with the Plug-In Grasshopper (version 1.0.0007, Robert McNeel & Associates, Seattle, WA 98103, USA) and ELISE (version 1.0.38, Synera GmbH, Bremen, Germany) as well as the software Synera (version 23.05, Synera GmbH, Bremen, Germany). Numerical results were obtained using the solver OptiStruct (Altair^®^ HyperWorks^®^ Version 2019, Altair Engineering, Inc., Troy, MI, USA).

### 2.1. Diatom-Inspired Dome Structures

Different dome structures inspired by diatoms were first constructed, before they were analyzed regarding their eigenfrequencies and mode shapes.

#### 2.1.1. Construction

**Figure 3:**
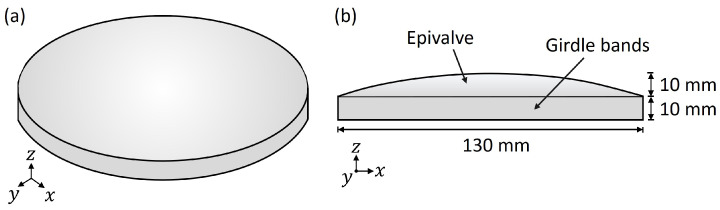
Basic dome structure model, i.e., ‘reference model’, in a 3D view (**a**) and a side view (**b**).

Different CAD (computer-Aided Design) shell models of dome structures were constructed within this study. All models were based on a general model layout similar to a diatom shell half. As shown in Figure 3, this basic model includes an upper convex surface (i.e., design space) and a lower cylindrical belt-like structure that was open (i.e., non-design space). The convex surface was created by the rotation of an interpolated curve and a ruled surface based on these leading curves. The following vertical extrusion of the surface edge created the lower cylinder.

The basic (reference) model was scaled in comparison to the approximate diameter of real diatoms

(1) $${⌀}_{diatom}=13\;\mu m$$

(2) $${⌀}_{model}=\eta \;{⌀}_{diatom}$$

The scale factor η was set to 10,000. However, since the focus of the study lied on the effect of structural patterns present in diatoms on eigenfrequencies and mode shapes, and studies show that eigenfrequencies scale with the factor 1/η [26,27], the difference in dimension is acceptable. In addition, the chosen model size permits a future manufacturing of the designed structures using 3D printing technologies for experimental studies.

**Figure 4:**
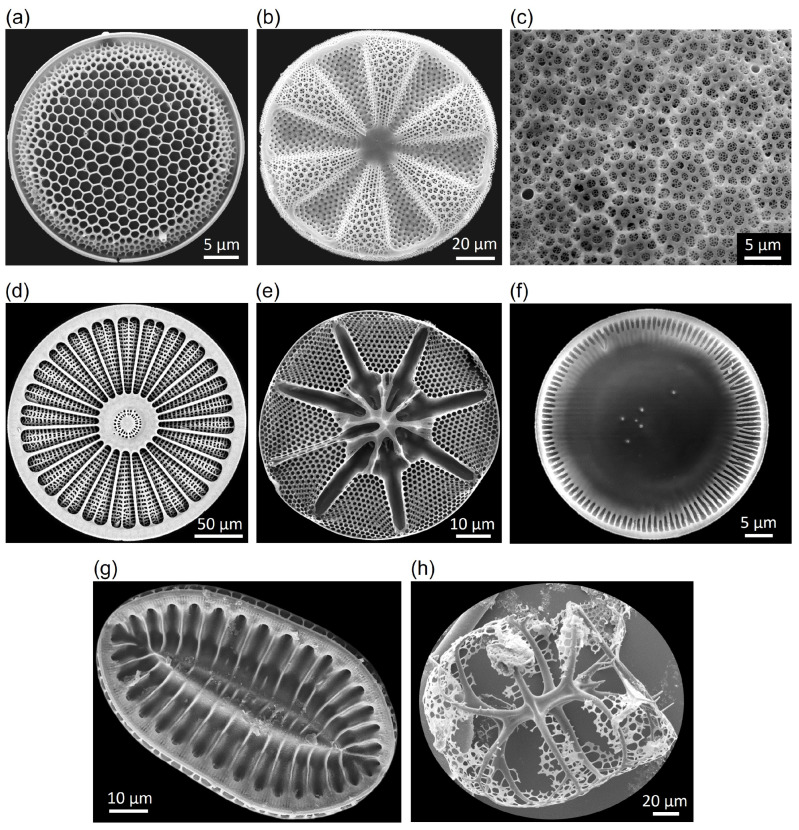
Scanning electron microscopic images from different diatoms showing comb patterns [(**a**) *Thalassiosira* sp., (**b**) *Actinoptychus* sp., (**c**) *Coscinodiscus wailesii*—a section from the epivalve with fractale combs), and stiffening rib structures, (**d**) *Arachnoidiscus* sp., (**e**) *Asteromphalos* sp., (**f**) *Cyclotella* sp., (**g**) *Surirella* sp.]. (**h**) shows a radiolarian. The images were made by L. Friedrichs/AWI (**a**–**f**), F. Hinz/AWI (**g**), and N. Niebuhr/AWI (**h**).

The design of the in total 48 dome structures was primarily inspired by microscopic images of different diatom species, whose frustules varied in shape and structural patterns. Aside from information provided by [28], microscopic images from the Alfred Wegener Institute Helmholtz Centre of Polar and Marine Research were taken as design inspiration. It was focused on regular and irregular comb patterns (e.g., *Thalassiosira* sp., *Actinoptychus* sp., *Coscinodiscus* sp.), stiffening rib structures (e.g., *Arachnoidiscus* sp., *Asteromphalus* sp., *Cyclotella* sp.), and (undulating) bulging segments (e.g., *Actinoptychus* sp.) (Figure 4a–f). In addition, various CAD models were designed by merging structural patterns of different diatom species (e.g., oval structures inspired by *Surirella* sp., Figure 4g) or considering other structural features visible in nature (e.g., branch-like hierarchical structures inspired by radiolarian, Figure 4h). The used software provided algorithms like ‘irregular point distribution’ or ‘Voronoi tessellation’, which allowed a systematic design of the complex diatom-inspired structures.

#### 2.1.2. Modal Analyses

Numerical finite element (FE) analyses were conducted to obtain the natural frequencies of the modeled dome structures. FE analysis involves a discretization of the geometry to be analyzed into smaller regions, i.e., finite elements. Each element is defined by a specific number of points (i.e., nodes). For each node, a polynomial function has to be differentiated to obtain a displacement result. The displacement within an element is approximated based on a linear combination of the nodal results. The solution of the finite number of differential equations of motion can be approximated using the virtual work principle. For more information it is referred to [29].

The present work deals with structural vibrations. Based on [30,31], the following equations of motion are used for an undamped free vibration system with multi-degrees of freedom (DOF):

(3) $$M\ddot{x}\left(t\right)+Kx\left(t\right)=0$$

where M and K are n×n (with *n* symbolizing the number of degrees of freedom of the entire structure) mass and stiffness matrices of the system and x and $\ddot{x}$ are n×1 vectors of the displacement and acceleration, respectively. The exact solution, in which $\hat{x}$ symbolizes the vibration amplitude vector and $\omega_{n}$ the *n*-th angular eigenfrequency, is:

(4) $$x\left(t\right)=\hat{x}\sin\left(\omega_{n}t\right)\;\;\;\;;\;\;\;\;n=1,2,3,\ldots$$

Inserting Equation (4) in Equation (3) accompanied by additional simplifications leads to the eigenvalue problem:

(5) $$\left(-{\omega_{n}}^{2}M+K\right)\hat{x}=0\;\;\;\;;\;\;\;\;n=1,2,3,\ldots$$

The roots of Equation (5) are the eigenvalues. In order to determine those eigenvalues, the determinant of the equations of motion is set equal to 0 (i.e., non-trivial solutions):

(6) $$\det(-{\omega_{n}}^{2}M+K)=0\;\;\;\;;\;\;\;\;n=1,2,3,\ldots$$

Equation (6) leads to the following *n*-th degree polynomial with the roots $\omega_{1}$, $\omega_{2}$, …, $\omega_{n}$ as the angular eigenfrequencies:

(7) $$a_{n}{\left({\omega_{n}}^{2}\right)}^{n}+a_{n-1}{\left({\omega_{n-1}}^{2}\right)}^{n-1}+\ldots +a_{1}\left({\omega_{1}}^{2}\right)+a_{0}=0\;\;\;\;;\;\;\;\;n=1,2,3,\ldots$$

The eigenvectors (i.e., mode shapes) can be determined by substituting the eigenvalues back into the eigenvalue problem. Each mode shape of a structure is associated with a specific eigenfrequency. Eigenfrequencies and mode shapes of complex structures can be determined using numerical modal analyses. Within the modal analysis results, the mode shapes are ordered by ascending frequency values. Thus, if eigenfrequencies are increased due to structural adaptation, the mode shapes can switch order (‘mode switching’).

In the present study, the eigenfrequencies (and mode shapes) of each dome structure were calculated by modal analyses using the FE method. The dome models were meshed with three-node shell elements (TRIAs) with six degrees of freedom per node to reproduce the complex shapes. A general element size of 1.0 mm was specified. Within a mesh study, the average element size was varied from 2.0 mm to 0.7 mm to demonstrate that an element size of 1.0 mm was adequate to obtain reliable numerical results. The mesh study was exemplarily applied to two dome structures.

For the conducted modal analyses, the translations of the lower nodes (i.e., the lower edge of each dome) were constrained (*x* = *y* = *z* = 0). In order to study the impact of the structural patterns on the eigenfrequencies, constant material properties were defined for all simulated structures. It was decided to assign the material properties listed in Table 1, i.e., AlSi10Mg, to each model, as the models might be manufactured out of aluminum in future experimental studies. In addition, the model mass was set to a constant value of 87 g to compare the impact of the different structural patterns on the eigenfrequencies. Thus, the shell thickness varied for each model.

The shell thickness, the model mass, the 1st eigenfrequency, and the 1st mode shape of each model were recorded.

Aside from the different dome structures, a model analysis of a simple base model visible in Figure 3 was conducted considered as the reference model. The mesh properties, the model assembly, and the boundary conditions corresponded to those defined for the complex dome structures. A shell thickness of 1.813 mm was set for the reference structure to obtain the specified model mass of 87 g.

The eigenfrequency deviation Δf comparing the eigenfrequency values from model 1, $f_{1}$, and model 2, $f_{2}$, were calculated as follows:

(8) $$\Delta f=\frac{f_{2}-f_{1}}{f_{1}}$$

### 2.2. Thickness Optimization of Dome Structures

In the previous chapter, different dome structures showing structural patterns visible in diatoms were studied regarding their eigenfrequencies and mode shapes. In the following studies, dome structures equipped with different rib patterns were optimized to maximize the 1st eigenfrequency and compared to the results of the previous chapter. The base structure as well as the mesh properties, the boundary conditions, and the material properties were equal to those defined for the previous study to obtain comparable structures. The structural mass was accordingly kept as a constant value of 87 g. Thickness optimizations were conducted with the objective to maximize the 1st eigenfrequency considering (a) regularly, and (b) irregularly distributed ribs on the top of the upper dome structure surface (i.e., the epivalve in Figure 3).

#### 2.2.1. Regularly Distributed Ribs

**Figure 5:**
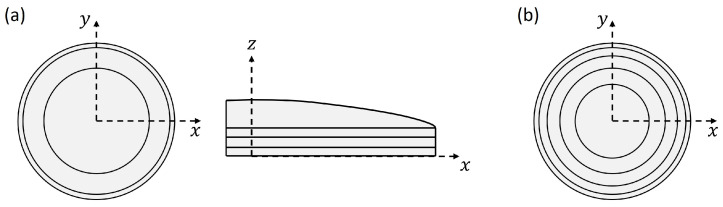
Subdivisions of the epivalve and the lower cylinder of the basic dome structure for the regularly distributed ribs (**a**). For the irregularly distributed ribs, the epivalve subdivisions are shown in (**b**).

Both the epivalve and the lower cylinder of the base structure were divided into three surfaces as visible in Figure 5a. For each of the resulting six dome surfaces, a constant shell thickness value was defined that was varied within the thickness optimization.

Regular honeycombs were distributed on the epivalve considering seven different comb spacing values of 5, 8, 12, 16, 20, 25, and 30 mm. Note that a large spacing value led to a low honeycomb density, i.e., large honeycombs.

Afterwards, 2D Voronoi tessellation based on a point distribution was used. Voronoi combs are polygons based on the intersection of lines created midway between the distributed points [32]. As a consequence, the Voronoi comb design is unique for a given point distribution.

Here, the irregularly shaped Voronoi combs were distributed on the epivalve and extruded in *z* direction by 4 mm (constant rib height) to form the ribs. Four different models were optimized with constant Voronoi comb spacing values of 5, 10, 15, and 20 mm. Both the thickness values of the six surfaces forming the basic dome structure and the rib thickness values were altered during the optimization process. The defined parameter ranges were 0.1 mm to 6.5 mm and 0.1 mm to 6.0 mm for the thickness values of the dome surfaces and the ribs, respectively. For all models, the optimization objective was to maximize the 1st eigenfrequency at a constant mass of 87 g.

#### 2.2.2. Irregularly Distributed Ribs

**Figure 6:**
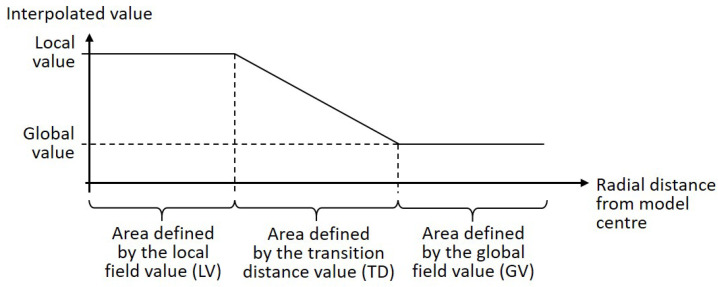
Illustration of the field function based on a local value and a global value, which are valid in certain areas within the model.

While the cylinder surface division into three surfaces was maintained (Figure 5)a, the epivalve was divided into five surfaces (Figure 5b). A field function (Figure 6) was used to define the point distribution on the top surface of the dome structure. The local field value was valid in the center of the top surface of the dome structure. Thus, the point distribution at the remaining surface area was defined by the global field value and the transition distance value. Finally, the Voronoi combs were created based on the irregular point distribution.

The optimization objective and mass constraint, as well as the rib height, and the definition of the parameter ranges for the thickness of the dome surfaces and the ribs followed the specifications in Section 2.2.1. The field parameters were defined in such a way that large combs appeared in the dome structure middle and small combs close to the border, since a comb size gradient from larger combs in the middle towards smaller combs at the border is present in many diatom frustules (e.g., *Thalassiosira* sp., Figure 4a). In addition, a similar gradient has been the result from already published structural optimizations [8]. The parameter value ranges considered to design the different rib patterns were 8 to 18 mm for the local field value, 10 to 30 mm for the transition distance, and 2 to 5 mm for the global field value.

For the model with the highest 1st eigenfrequency, the constant rib height was altered. Height values of 3.0 mm, 4.5 mm, and 5.0 mm were defined to check whether different rib heights would lead to a higher 1st eigenfrequency. For this, thickness optimizations with the above mentioned settings were again performed.

In total, 14 different dome models with irregularly distributed ribs were calculated. The results were compared to those obtained for the regularly distributed ribs and to the diatom-inspired dome models considering Equation (8) to compute the eigenfrequency deviations.

## 3. Results

This section presents the results of the conducted studies.

### 3.1. Diatom-Inspired Dome Structures

#### 3.1.1. Construction

**Figure 7:**
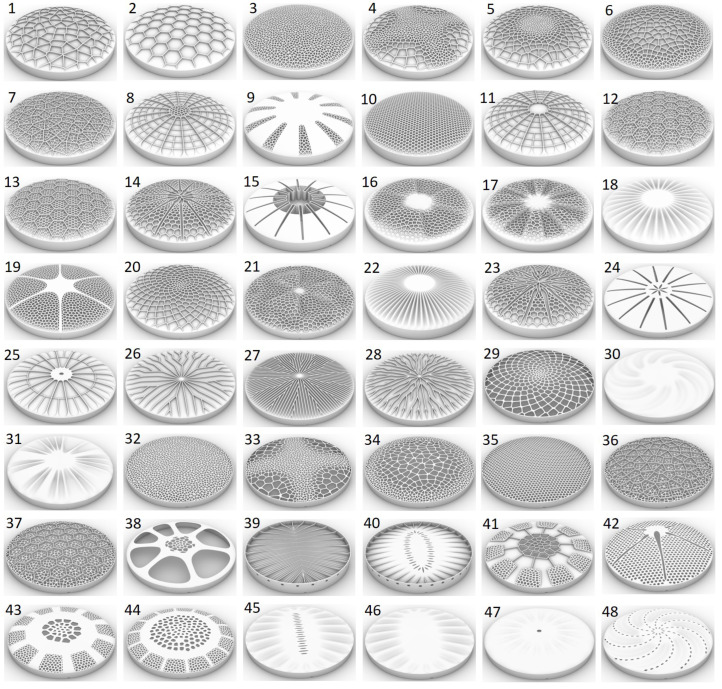
CAD constructed dome structures inspired by diatoms.

48 frustule models with different structural patterns have been constructed as visible in Figure 7. Generally, the models were characterized by (a) combs (e.g., models 1, 20, and 33), (b) stiffening ribs (e.g., models 8, 11, and 25), (c) undulating bulging segments (e.g., models 16, 30, and 46), and (d) models with varying structural patterns (e.g., models 9, 15, and 38).

The comb models showed irregular (Voronoi) and regular (honey-)comb structures in small and large cell sizes or with varying cell sizes due to the definition of cell size gradients. Some comb structures were constructed as ribs on top of a simple shell structure, others formed the dome structure itself. Aside from comb structures, different types of stiffening structures in form of radially oriented ribs were designed. Some models showed undulating bulges varying in geometry, size, and number of bulges. In addition, structural hierarchies were present in some models, e.g., hierarchical rib layouts or fractale honeycombs (i.e., ‘each comb cell was filled with smaller comb cells’). The majority of the designed structural patterns were characterized by radial symmetry, but several domes had different asymmetrical structures, which can also be found in diatoms.

#### 3.1.2. Modal Analyses

**Figure 8:**
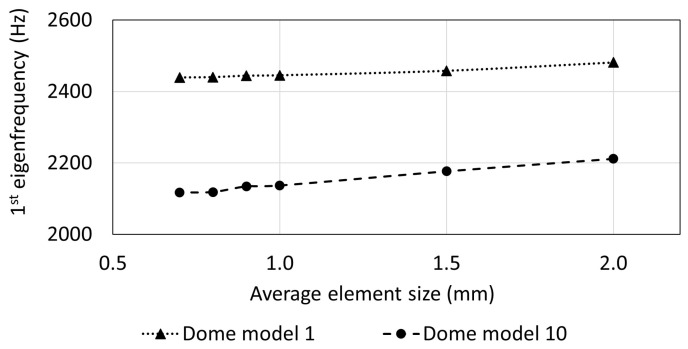
1st eigenfrequency depending on the element size for the dome models 1 and 2.

**Figure 9:**
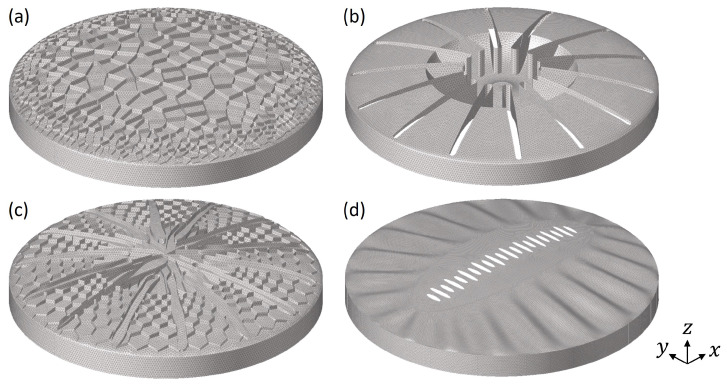
FE meshes of the studied dome structures (**a**) model 6; (**b**) model 15; (**c**) model 23, and (**d**) model 45.

The initially conducted mesh study showed that the 1st eigenfrequencies did almost not change for models meshed with elements that were smaller than the chosen element size of 1.0 mm (Figure 8). However, very small structural patterns were meshed with smaller element sizes between 0.7 mm to 0.9 mm. The average number of element per model was 89,000. Figure 9 exemplarily shows the fine FE meshes of four different models.

**Figure 10:**
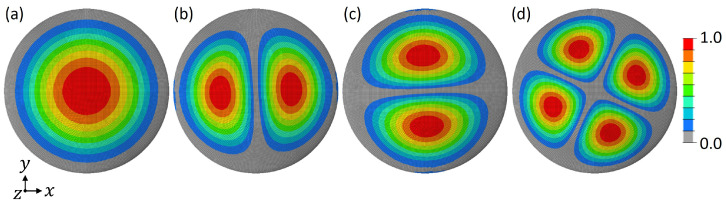
Top view of the 1st (**a**), 2nd (**b**), 3rd (**c**), and 4th (**d**) mode shape of the reference model. The colouring represents the absolute normalized vibration amplitude.

Regarding the reference model, the obtained 1st eigenfrequency $f_{1,\mathrm{ref}}$ was 2799 Hz. The first four mode shapes are displayed in Figure 10.

Table 2 lists the minimum value, the maximum value, and the average value of the shell thickness, the mass, and the 1st eigenfrequency among all 48 models. While the model mass was constant as initially stated, the shell thickness varied from 0.92 mm to 5.51 mm. The lowest 1st eigenfrequency was 660 Hz, while the highest value was 4139 Hz, i.e., more than six times higher than the lowest eigenfrequency value.

**Figure 11:**
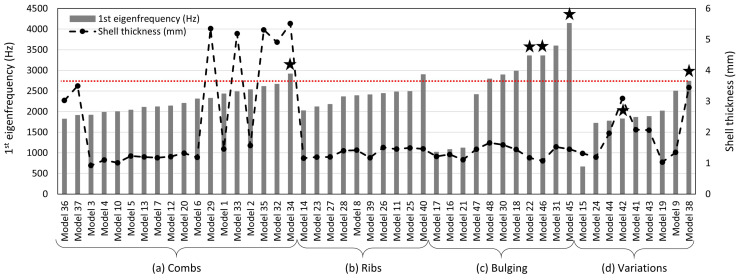
1st eigenfrequency and shell thickness of all dome models sorted by the structural patterns (**a**) combs, (**b**) ribs, (**c**) bulging, and (**d**) variations, and organized by increasing 1st eigenfrequency. The red, dotted line shows the 1st eigenfrequency of the reference structure. The six models showing a 1st mode shape similar to the 2nd mode shape of the reference model, i.e., two bulges, are marked with a star above the frequency bar.

In Figure 11, the 1st eigenfrequency and the shell thickness of all models are plotted. The models are organized by the structural patterns (a) combs, (b) ribs, (c) bulging, and (d) geometrical variations, and ordered by their 1st eigenfrequency values.

Regarding the shell thickness, the majority of the models show values around 1.0 mm to 1.5 mm, while only some models had clearly higher shell thickness values up to more than 5 mm. However, the mass always remained constant.

As for the 1st eigenfrequency, four models had comparably low 1st eigenfrequencies of less than 700 Hz to slightly above 1000 Hz. The 1st eigenfrequencies of the majority of the dome structures laid within a range of about 1800 Hz to 2500 Hz, which is below the 1st eigenfrequency of the reference structure ($f_{1,\mathrm{ref}}$ = 2799 Hz). Despite one model, all models of the geometrical groups combs, ribs, and variations had lower 1st eigenfrequencies than the reference structure. On the other side, the majority of the bulging-models showed very high 1st eigenfrequency values up to more than 4000 Hz.

In total, eight models had a higher 1st eigenfrequency than the reference structure. Four of them (marked with a star in Figure 11) showed a mode shape switch compared to the reference structure, i.e., the 1st mode shape was similar to the 2nd mode shape of the reference structure. (cf., Figure 10). Thus, the frequency of the reference structure’s 1st mode shape was shifted to a higher frequency value. The remaining two models with a mode switch were ‘geometrical variations’-models and had lower 1st eigenfrequencies than the reference model. For the remaining dome models, the studied mode shapes were similar to those of the reference structure and did also appear in the same order.

**Figure 12:**
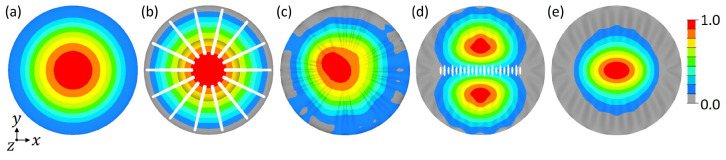
1st mode shapes of diatom-inspired dome structures. Displayed are the models 10 (**a**), 15 (**b**), 31 (**c**), 46 (**d**), and 45 (**e**). The colouring represents the absolute normalized vibration amplitude.

Figure 12 shows exemplarily the 1st mode shapes of five models. Models with comparably lower 1st eigenfrequency values (Figure 12a,b) tend to show a 1st mode shape similar to the reference model, i.e, one large bulge that covers (almost) the whole model surface. However, many models with comparably high 1st eigenfrequencies showed either a 1st mode shape similar to the 2nd mode shape of the reference model (i.e., mode shape switch occurred, e.g., Figure 12d), or a deformed 1st mode shape (e.g., Figure 12c), or a mode shape that covers only a part of the model surface (e.g., Figure 12e).

**Figure 13:**
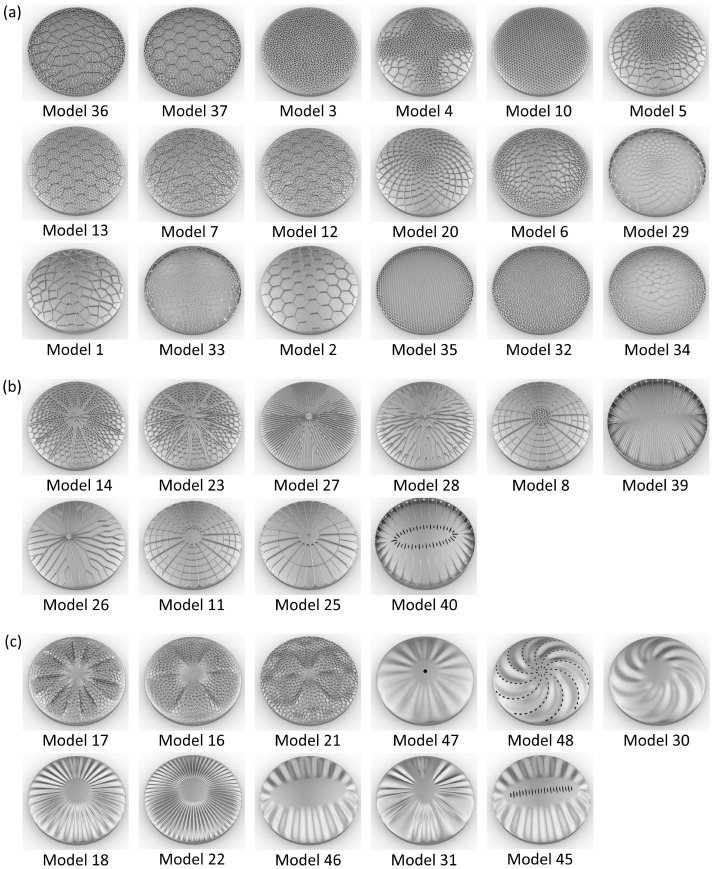

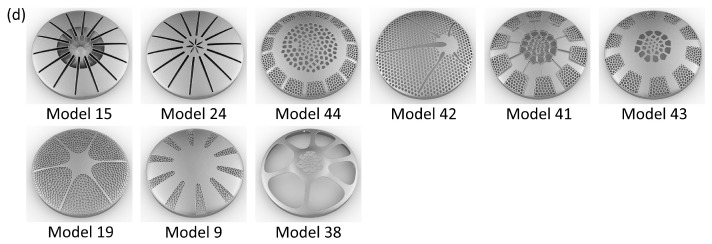
Modelled dome structures sorted by (**a**) combs, (**b**) ribs, (**c**) bulging, and (**d**) geometrical variations, and ordered by increasing 1st eigenfrequency.

The following statements can be concluded based on indications of the results:

(a) Combs (Figure 13a)
  - For models showing a constant comb size (both honeycombs and Voronoi combs): a larger comb size tends to increase the 1st eigenfrequency by about 27% (models 10 vs. 2, and 3 vs. 1)
  - For models with regular and irregular combs (no fractale combs): the 1st eigenfrequency seems to be significantly higher (>25%) if the comb pattern forms the structure itself compared to comb patterns applied as ribs to a simple dome structure (models 10 vs. 35, 3 vs. 32, 4 vs. 33, and 6 vs. 34). However, for models with fractale honeycombs or Voronoi combs, applying the comb pattern as ribs to a simple dome surface shows an increase of the 1st eigenfrequency by 11% to 14% (models 36 vs. 7, and 37 vs. 12)
  - Generally, smaller combs close to the model’s border and larger combs in the middle tends to increases the 1st eigenfrequency (increase of 21% for model 3 vs. 6)
  - For models with a constant comb size, the comb unit geometry (regular honeycomb, irregular Voronoi, or fractale combs) seems to almost not affect the 1st eigenfrequency (deviations < 5%; models 1 vs. 2, 3 vs. 10, and 32 vs. 35)
(b) Ribs (Figure 13b)
  - A lower number of stiffening ribs tends to increase the 1st eigenfrequency by more than 9% (models 27 vs. 26, and 27 vs. 28)
  - Placing voids in the model’s centre, especially an irregular void pattern, in combination with stiffening the model’s border apparently increases the 1st eigenfrequency, here the increase was 20% (model 39 vs. 40)
  - Small geometrical adaptations that almost do not affect the mass, or that are not close to the model’s centre are likely to almost not alter the 1st eigenfrequency (models 14 vs. 23, 11 vs. 25, and 11 vs. 8)
(c) Bulging (Figure 13c)
  - Irregular or deformed undulating bulges seem to increase the 1st eigenfrequency by about 20% (models 18 vs. 31, and 47 vs. 30)
  - The results indicate that the smaller scaled the bulging pattern, the higher the 1st eigenfrequency (increases of more than 12% for models 47 vs. 18, and 18 vs. 22)
  - Small-scaled bulges close to the border that increase the stiffness in combination with an irregular embossing shape in the model’s centre tends to lead to a very high eigenfrequency, especially if the mass in the model’s centre can be reduced (1st eigenfrequency increase of 23% for model 46 vs. 45)
  - The number of radial symmetric undulating segments appears to only alter the 1st eigenfrequency less than 6% (models 16 vs. 21, and 17 vs. 16)
  - Small voids distributed over the whole model area seem to hardly affect the 1st eigenfrequency (model 30 vs. 48)
(d) Geometrical Variations (Figure 13d)
  - The results indicate that stiff borders are at least equally important to increase the freuency, so that models with stiff borders and without voids in the middle can show higher eigenfrequencies (1st eigenfrequency increase of 41% and 33% for the models 9 vs. 44 and 9 vs. 43, respectively)

### 3.2. Thickness Optimization of Dome Structures

**Figure 14:**
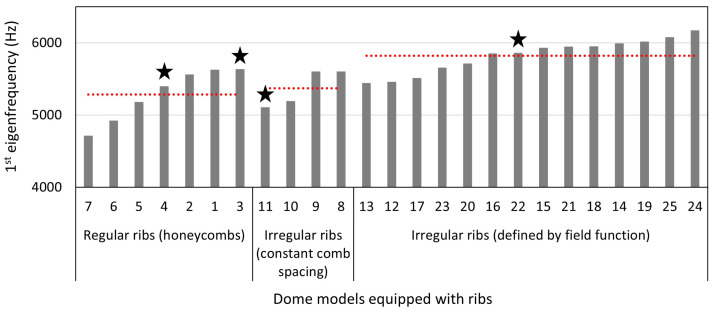
1st eigenfrequency of the dome models equipped with ribs obtained by thickness optimizations. For the three geometrical groups regular ribs, irregular ribs with constant comb spacing, and irregular ribs defined by field functions, the models are ordered by increasing 1st eigenfrequency. In addition, for each group, the red, dotted line shows the average 1st eigenfrequency value. The four models showing a 1st mode shape similar to the 2nd mode shape of the reference model, i.e., two bulges, are marked with a star above the frequency bar.

25 dome structures with rib patterns were modeled. The defined geometrical characteristics, and the 1st eigenfrequency and mode shapes obtained by the thickness optimizations are listed in Table 3. In Figure 14, the models are grouped by their geometrical characteristics and ordered by their 1st eigenfrequencies. All models had a constant model mass of 87 g to be compared to the diatom-inspired dome structures analyzed in the previous study part. The 1st eigenfrequency values were between 4708 Hz and 6165 Hz, and the average 1st eigenfrequency values were 5286 Hz for the regular honeycombs, 5372 Hz for the irregular Voronoi ribs with constant comb spacing, and 5822 Hz for the irregular ribs defined by the field functions. Thus, the 1st eigenfrequency generally increased with rising degree of structural irregularities.

**Figure 15:**
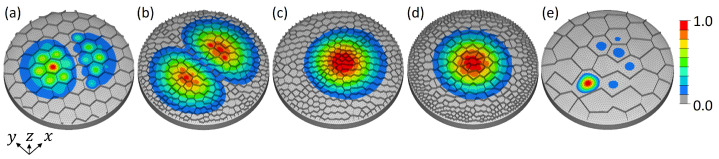
1st mode shapes of dome structures equipped with ribs. Displayed are the models 4 (**a**), 22 (**b**), 8 (**c**), 24 (**d**), and 11 (**e**). The colouring represents the absolute normalized vibration amplitude.

Regarding the 1st mode shapes, all models aside from four models (marked with a star in Figure 14) showed a 1st mode shape similar to that of the reference model (cf., Figure 10a), i.e., one large bulge. Nevertheless, all 1st eigenfrequencies were significantly higher than the 1st eigenfrequency of the reference structure ($f_{1,\mathrm{ref}}$ = 2799 Hz). Figure 15 exemplarily displays the 1st mode shapes of five models. For the models 8 and 24 (Figure 15c,d), which showed comparably high $f_{1,\mathrm{ref}}$ eigenfrequencies, the $f_{1,\mathrm{ref}}$ mode shape was similar to that of the reference structure. However, the one large bulge did not cover the whole model, as for the reference structure, but only a small part of the structure’s surface was deflected. Three of the four models having a 1st mode shape that differed from that of the reference model are visible in Figure 15a,d,e (models 4, 22, and 11). Although the $f_{1,\mathrm{ref}}$ mode shapes varied strongly compared to the reference model, the $f_{1,\mathrm{ref}}$ eigenfrequencies were not the highest among the here studied models (model 24 showed a higher $f_{1,\mathrm{ref}}$ eigenfrequency). For the models 4 and 22, the $f_{1,\mathrm{ref}}$ mode shapes were similar to the 2nd mode shape of the reference model (cf., Figure 10b). Also here, the two bulges did not cover the whole model surface, but were smaller or even deformed, i.e., non-symmetrical. The last model (model 11) had a very different 1st mode shape (Figure 15e).

### 3.3. Comparison

**Figure 16:**
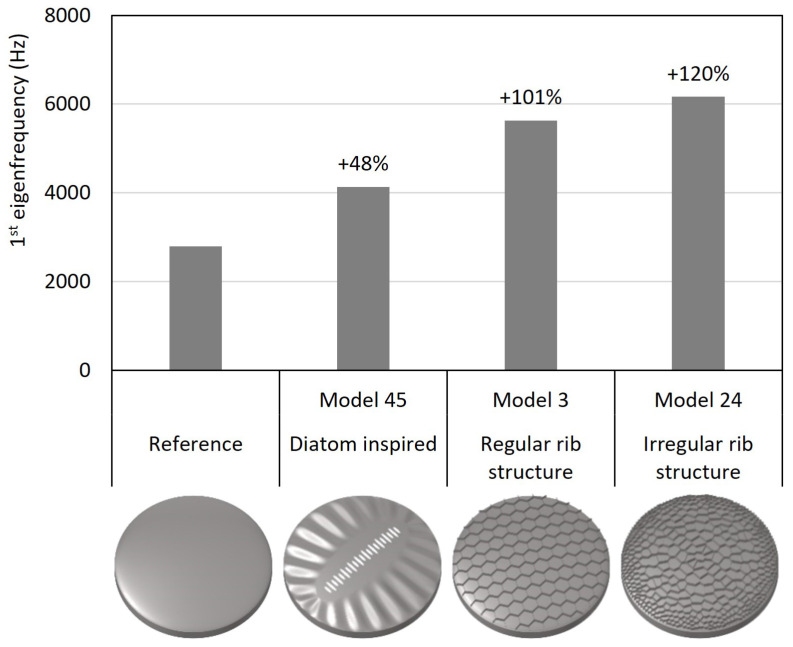
Comparison of the 1st eigenfrequency of the reference dome structure to the models with the highest 1st eigenfrequency among the diatom-inspired dome structures, the dome structures equipped with regular ribs, and the dome structures stiffened by applying irregular rib patterns. The 1st eigenfrequency increase compared to the reference model is given above each bar. All models had the same general dimensions, the same material properties, and the same mass.

All studied models coincided in their general dimensions, material properties, and structural mass. In Figure 16, the models with the highest 1st eigenfrequencies among the diatom-inspired models and the models with regular and irregular rib patterns are compared to the reference model. While the diatom-inspired models showed 1st eigenfrequency increases up to 48% compared to the reference model, the dome structures equipped with rib patterns had far higher 1st eigenfrequency increases of up to 101% for the regular ribs and up to 120% for the irregular ribs.

**Figure 17:**
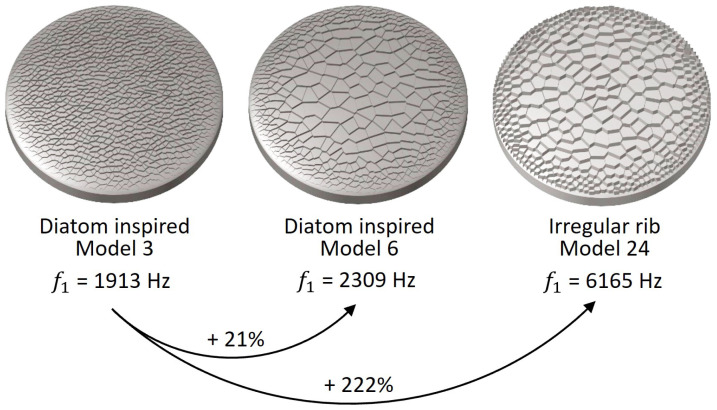
1st eigenfrequency increase of the diatom-inspired model 6 and the thickness optimization model 24 compared to the diatom-inspired model 3. While the latter shows uniformly sized combs, the other two models are characterized by a comb size gradient from smaller combs at the border and larger combs in the middle.

Exemplarily, three different models with comb patterns were compared among each other (Figure 17). The diatom-inspired model 6 was characterized by a comb size gradient with large combs in the middle and small combs close to the border. The model showed a 1st eigenfrequency increase of 21% compared to diatom-inspired model 3 which had only uniformly sized combs. Among the optimized models were also several models showing a comb size gradient similar to that of the diatom-inspired model 6 like for example model 24. Compared to diatom-inspired model 3, the 1st eigenfrequency was increased by 222% for thickness optimization model 24. It has to be noted that the latter had different thickness values assigned to each element, while diatom-inspired models 3 and 6 showed a constant thickness value.

## 4. Discussion

### 4.1. Diatom-Inspired Dome Structures

Different diatom-inspired dome structures have been constructed. In contrast to other published simulation studies on diatom shells, structural complexity in diatom shells was considered here, resulting in different models based on regular and irregular combs, stiffening patterns, and bulging designs. The models were scaled by 10,000 in comparison to the approximate diameter of real diatoms (130 μm vs. 130 mm), so that manufacturing using the 3D printing technology and vibration experiments could be addressed in continuative studies.

The conducted modal analyses resulted in 1st eigenfrequency values and mode shapes for all diatom-inspired models. The initial mesh study indicated that the chosen element size was adequate, because the 1st eigenfrequency did hardly vary with further decreasing element size, i.e., with refining the mesh. While the reference model had a 1st eigenfrequency of 2799 Hz, the 1st eigenfrequencies of the diatom-inspired models were between 660 Hz and 4139 Hz. The majority of the models had smaller 1st eigenfrequency values than the reference model, although they showed structural patterns generally leading to high stiffness at low mass [33]. As a high stiffness usually results in a high 1st eigenfrequency, higher 1st eigenfrequency values have been expected. However, the study indicates that structural patterns cannot simply be applied to a certain technical problem to improve the mechanical properties. Instead, the structural patterns have to be adapted to the specific design space and boundary conditions to efficiently maximize the eigenfrequencies, which is why in the second part of this study, comb patterns were exemplarily optimized to increase the 1st eigenfrequency.

For almost all models, the 1st mode shape were similar to that of the reference model, despite the different structural patterns applied and the varying eigenfrequency values. Yet, some models showed a mode switch. The results indicate that a mode switch is likely to occur at comparably high eigenfrequency values. In addition, variations in the appearance of a mode shape, e.g., decreasing the size of the single large bulge of the 1st mode shape, is likely to come along with increased 1st eigenfrequency, which has also been stated in other studies, e.g., [24]. However, this does not generally apply for all models, because also mode switch at a comparably low eigenfrequency value was recorded. In addition, another model with a comparably high 1st eigenfrequency showed no mode switch. These findings were not studied further, because the focus of the present study lied on how the diatom-inspired structural patterns affect the eigenfrequencies.

Based on the different diatom-inspired models studied, general statements of how structural patterns can significantly affect eigenfrequencies have been expressed. These statements are expected to support the design process of structures with high eigenfrequencies. However, it has to be noted that the statements are based on a limited number of models, which is why detailed studies on structural patterns strongly affecting the eigenfrequencies are recommended. In addition, combining structural patterns with parameter studies and structural optimizations, in which the boundary conditions and optimization objectives are clearly defined, is expected to further increase the eigenfrequencies and to lead to very promising results.

As already mentioned, the diatom-inspired models were scaled with a factor of η = 10,000. It has been proposed that eigenfrequencies scale with 1/η [26,27]. Thus, the here simulated eigenfrequencies were scaled to obtain the eigenfrequencies for models in the dimensions of real diatoms. As the performance of eigenfrequency scaling is a common procedure for constant material properties and linear behaviour, it is expected that the scaling does not particularly affect the structural patterns present in the studied dome models.

In addition to the scale factor 1/η, a scale factor ϵ was defined to consider the different material properties with the aim to compare the obtained results to already published data on diatom eigenfrequencies. While in the present study, aluminum was specified as material, the Young’s modulus, density, and Poisson’s ratio vary from the values obtained for real diatom shells published by [18] (Table 4).

Following the general definition of an eigenfrequency depending on stiffness and mass, the scale factor ϵ was defined as:

(9) $$\epsilon =\sqrt{\frac{E_{p}/E_{c}}{\rho_{p}/\rho_{c}}}$$

with $E_{p}$ and $E_{c}$ representing the Young’s modulus of the present study and obtained by [18], respectively, and $\rho_{p}$ and $\rho_{c}$ symbolizing the material densities, respectively.

Thus, each eigenfrequency of a diatom-inspired dome models $f_{p}$ was scaled to receive an estimated 1st eigenfrequency value of a diatom shell $f_{\mathrm{diatom}}$:

(10) $$f_{\mathrm{diatom}}=\frac{1}{\eta }\frac{1}{\epsilon }f_{p}$$

The eigenfrequency scaling led to 1st eigenfrequency values between 3 MHz and 20 MHz, which were within the range of diatom eigenfrequencies obtained in other studies [18,19]. Thus, the simulation models can be seen as resilient.

### 4.2. Thickness Optimization of Dome Structures

After showing that diatom-inspired comb patterns tend to increase the 1st eigenfrequency, the efficiency of comb patterns in manipulating the eigenfrequencies was studied in detail. The simple dome models were equipped with rib patterns based on regular and irregular combs. Subsequent thickness optimizations aimed at maximizing the 1st eigenfrequency while maintaining the model mass. As the boundary conditions, material properties, simulation set-up, and model mass corresponded to that of the diatom-inspired dome models, all resulting designs could be compared among each other.

The thickness optimizations resulted in different dome models with significantly higher 1st eigenfrequencies than the reference model. Even the regular comb patterns led to very high eigenfrequencies. This indicates the approach of optimizing the shell thickness of both the rib pattern, and the base structure itself to be very efficient in increasing eigenfrequencies. Regarding the mode shapes of the simulated models, mode switch compared to the reference model occurred only in a few models. Similar to the diatom-inspired dome structures, the models showing a mode switch did not have necessarily the highest 1st eigenfrequency, which has already been discussed in the previous chapter.

Among the studied models, the highest 1st eigenfrequencies were obtained by using irregular comb patterns and applying comb size gradients. The best models showed small combs close to the border and large combs in the middle. This corresponds to the results obtained by [8].

Thus, it can be concluded that the combination of (1) choosing structural patterns inspired by diatoms that are expected to lead to high stiffness and high eigenfrequencies and (2) conducting structural optimizations to discover an optimum shape of the pattern considering the boundary conditions and design objectives is a promising procedure in designing structures with high eigenfrequencies.

### 4.3. Comparison

The final comparison of the models showed that a significant manipulation of the 1st eigenfrequency values in comparison to the reference model was possible. While the 1st eigenfrequency increased up to 48% for the diatom-inspired models, it rose by up to 101% and 120% for the regular and irregular rib patterns, respectively.

The efficiency of the here proposed method to design diatom-inspired structures with high eigenfrequency was exemplarily demonstrated for the application of comb patterns. Among the diatom-inspired models, a significant eigenfrequency increase due to implementing a comb size gradient in comparison to uniformly sized combs was recorded. Thus, the structural feature of comb size gradients, which can be often observed in diatoms (e.g., *Thalassiosira* sp., Figure 4a), has a strong impact on the eigenfrequencies. However, in a first step, the structural pattern was solely applied to the simple dome structure. Boundary conditions and design objectives were considered. In a second step, different comb size gradients were tested in combination with a thickness optimization. Here, the boundary conditions and design objectives were implemented into the design process. The resulting 1st eigenfrequency was much higher compared to the previous model, and also compared to the reference structure. Thus, the studied two-step design process is an an efficient method to design diatom-inspired lightweight solutions with high eigenfrequencies.

## 5. Conclusions

We have constructed numerous complex dome structures inspired by diatoms. The implemented structural features were mostly based on regular and irregular combs, rib patterns, and embossing patterns. The 1st eigenfrequencies and mode shapes of all models were numerically studied detecting several diatom-inspired structural features to be efficient in manipulating eigenfrequencies. Comb patterns were afterwards studied in detail and combined with thickness optimizations to further increase eigenfrequencies. The design process of (1) selecting diatom-inspired structural patterns having a strong impact on eigenfrequencies, and (2) adapting them considering the boundary conditions and design objectives in combination with thickness optimizations is an efficient method to design diatom-inspired lightweight solutions with high eigenfrequencies.

## Figures and Tables

**Table 1:** Material properties of the aluminum alloy AlSi10Mg assigned to the models studied.

Property	Value
Young’s modulus, *E* (MPa)	75,000
Shear modulus, *G* (MPa)	28,195
Poisson’s ratio, ν (−)	0.33
Density, ρ (kg $m^{3}$)	2700

**Table 2:** Among all studied dome models, the minimum value, the maximum value, and the average value of the shell thickness, the mass, and the 1st eigenfrequency are listed.

	Shell Thickness (mm)	Mass (g)	1st Eigenfrequency (Hz)
MIN	0.92	87.00	660
MAX	5.51	87.08	4139
AVERAGE	1.92	87.02	2291

**Table 3:** Geometrical characteristics and optimization results for the thickness optimization study of dome structures. Based on the rib distribution type, the comb spacing/parameter values (GV: global value, LV: local value, TD: transition distance), and the rib height the models were designed. The listed 1st mode shapes are those of the reference model. ‘Mode X’ indicated that the mode shape was not similar to one of the studied mode shapes of the reference model.

Model Number	Rib Distribution	Comb Spacing Value/Parameter Values (mm)	Rib Height (mm)	1st Eigenfrequency (Hz)	1st Mode Shape
1	regular	5	4.0	5621	Mode 1
2	regular	8	4.0	5557	Mode 1
3	regular	12	4.0	5631	Mode 2
4	regular	16	4.0	5392	Mode 2
5	regular	20	4.0	5176	Mode 1
6	regular	25	4.0	4919	Mode 1
7	regular	30	4.0	4708	Mode 1
8	irregular	5	4.0	5599	Mode 1
9	irregular	10	4.0	5597	Mode 1
10	irregular	15	4.0	5188	Mode 1
11	irregular	20	4.0	5103	Mode X
12	irregular	GV = 5; LV = 15;	4.0	5453	Mode 1
		TD = 10			
13	irregular	GV = 5; LV = 18;	4.0	5438	Mode 1
		TD = 20			
14	irregular	GV = 3; LV = 8;	4.0	5987	Mode 1
		TD = 20			
15	irregular	GV = 3; LV = 10;	4.0	5927	Mode 1
		TD = 20			
16	irregular	GV = 3; LV = 12;	4.0	5846	Mode 1
		TD = 20			
17	irregular	GV = 3; LV = 16;	4.0	5507	Mode 1
		TD = 20			
18	irregular	GV = 3; LV = 8;	4.0	5944	Mode 1
		TD = 25			
19	irregular	GV = 3; LV = 8;	4.0	6011	Mode 1
		TD = 30			
20	irregular	GV = 3; LV = 15;	4.0	5706	Mode 1
		TD = 25			
21	irregular	GV = 2; LV = 8;	4.0	5942	Mode 1
		TD = 20			
22	irregular	GV = 4; LV = 8;	4.0	5857	Mode 2
		TD = 20			
23	irregular	GV = 3; LV = 8;	3.0	5649	Mode 1
		TD = 30			
24	irregular	GV = 3; LV = 8;	4.5	6165	Mode 1
		TD = 30			
25	irregular	GV = 3; LV = 8;	5.0	6072	Mode 1
		TD = 30			

**Table 4:** Material properties of the aluminum alloy AlSi10Mg assigned to the models of the present study compared to the material properties assigned to diatom models studied by Cvjetinovic et al. [18].

Property	Present Study	Cvjetinovic et al. [18]
Young’s modulus, *E* (MPa)	$E_{p}$ = 75,000	$E_{c}$ = 15,000
Density, ρ (kg $m^{3}$)	$\rho_{p}$ = 2700	$\rho_{c}$ = 2300
Poisson’s ratio, ν (−)	0.33	0.17

## Data Availability

Data are contained within the article.
